# Sensory neuronal sensitisation occurs through HMGB-1–RAGE and TRPV1 in high-glucose conditions

**DOI:** 10.1242/jcs.215939

**Published:** 2018-07-26

**Authors:** Samuel M. Bestall, Richard P. Hulse, Zoe Blackley, Matthew Swift, Nikita Ved, Kenneth Paton, Nicholas Beazley-Long, David O. Bates, Lucy F. Donaldson

**Affiliations:** 1 School of Life Sciences, The Medical School QMC, University of Nottingham, Nottingham NG7 2UH, UK; 2 Arthritis Research UK Pain Centre, The Medical School QMC, University of Nottingham, Nottingham NG7 2UH, UK; 3 Cancer Biology, School of Clinical Sciences, University of Nottingham, Nottingham NG7 2UH, UK; 4 Institute of Ophthalmology, 11-43 Bath St, London EC1V 9EL, UK

**Keywords:** HMGB1, RAGE, Nociceptor, Sensitisation, Vascular endothelial growth factor, Diabetes

## Abstract

Many potential causes for painful diabetic neuropathy have been proposed including actions of cytokines and growth factors. High mobility group protein B1 (HMGB1) is a RAGE (also known as AGER) agonist whose levels are increased in diabetes and that contributes to pain by modulating peripheral inflammatory responses. HMGB1 enhances nociceptive behaviour in naïve animals through an unknown mechanism. We tested the hypothesis that HMGB1 causes pain through direct neuronal activation of RAGE and alteration of nociceptive neuronal responsiveness. HMGB1 and RAGE expression were increased in skin and primary sensory (dorsal root ganglion, DRG) neurons of diabetic rats at times when pain behaviour was enhanced. Agonist-evoked TRPV1-mediated Ca^2+^ responses increased in cultured DRG neurons from diabetic rats and in neurons from naïve rats exposed to high glucose concentrations. HMGB1-mediated increases in TRPV1-evoked Ca^2+^ responses in DRG neurons were RAGE- and PKC-dependent, and this was blocked by co-administration of the growth factor splice variant VEGF-A_165_b. Pain behaviour and the DRG RAGE expression increases were blocked by VEGF-A_165_b treatment of diabetic rats *in vivo*. Hence, we conclude that HMGB1–RAGE activation sensitises DRG neurons *in vitro*, and that VEGF-A_165_b blocks HMGB-1–RAGE DRG activation, which may contribute to its analgesic properties *in vivo*.

## Abstract (precis)

**Summary:** In high-glucose conditions, nociceptive neurons are sensitised through the actions of HMGB1 acting though RAGE and PKC. This sensitisation is blocked by a VEGF-A splice variant.

## INTRODUCTION

Diabetic neuropathy affects up to 50% of diabetic patients (Obrosova, 2009), and results from changes in the peripheral sensory nerve microenvironment due to microvasculopathy and direct actions of high glucose on peripheral sensory neurons. Sensory neurons are particularly susceptible to hyperglycaemic damage as they lack insulin-regulated glucose uptake (Tomlinson and Gardiner, 2008). Peripheral sensory fibre damage in nerve trunks results in local inflammatory responses and the development of neuropathic pain, including symptoms of allodynia and hyperalgesia, in experimental models of diabetes and during naturally occurring diabetes (Calcutt et al., 2008).

Long-standing hyperglycaemia results in the formation and accumulation of advanced glycation end products (AGEs) (Singh et al., 2014), which can activate the receptor for AGE (RAGE; also known as AGER) and cause neuronal damage. Other RAGE ligands, such as the inflammatory cytokine high-mobility group box-protein 1 (HMGB1, also known as amphoterin), can act more rapidly to activate RAGE (Saleh et al., 2013), and may contribute to pain through modulation of neuro-inflammatory responses (Maeda et al., 2013). RAGE expression is increased in peripheral neurons in traumatic (Allette et al., 2014) and diabetic neuropathy (Juranek et al., 2013), and RAGE neutralisation inhibits neuropathic pain (Brederson et al., 2016). The mechanism(s) of RAGE signalling on sensory neuronal sensitisation are unknown.

HMGB1 levels are increased in the plasma of diabetic patients (Devaraj et al., 2009), and HMGB1 is implicated in chronic pain (Agalave and Svensson, 2015) associated with arthritis (Ke et al., 2015), and traumatic (Allette et al., 2014; Feldman et al., 2012; Nakamura et al., 2013) and chemotherapy-induced neuropathic pain (Nishida et al., 2016), probably through RAGE activation (Allette et al., 2014). HMGB1 sensitises and activates sensory neurones in a manner that would lead to altered nociceptive behaviour (Feldman et al., 2012), but the mechanisms through which these changes occur are unknown.

Transient receptor potential (TRP) channels such as TRPA1 and TRPV1 are implicated in both the pain (Cui et al., 2014; Pabbidi et al., 2008; Wei et al., 2009) and neuronal damage in diabetic neuropathy (Koivisto et al., 2012). The pain and neuronal damage in diabetic neuropathy are related to the decrease in expression of vascular endothelial growth factor-A (VEGF-A) (Jerić et al., 2017; Pawson et al., 2010), a family of growth factors with effects on both vascular and neuronal systems (Carmeliet and Storkebaum, 2002). We previously showed that pain and neuronal activation associated with the VEGF-A splice variant VEGF-A_165_a are mediated through effects on TRPV1 (Hulse et al., 2014). These effects can be reversed by the alternatively spliced VEGF-A_165_b isoform (Harper and Bates, 2008), which also decreases neuropathic pain behaviours in diabetic rats (Hulse et al., 2015).

In these studies we tested the hypotheses that: (1) hyperglycaemia is associated with increased peripheral HMGB1 expression in diabetic rats when mechanical and thermal hypersensitivity is present, (2) HMGB1 sensitises neurons through actions on TRPV1, (3) high-glucose conditions alter TRPV1-mediated signalling in sensory neurons through an HMGB1–RAGE–PKC-dependent mechanism, and (4) that the effects of HMGB1 on sensory neurons can be reversed by VEGF-A_165_b treatment.

## RESULTS

**Fig. 1.**
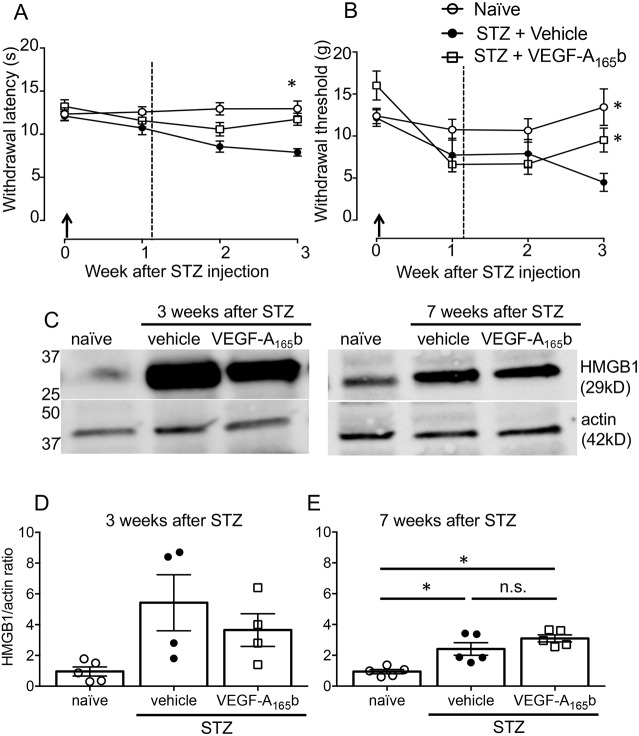
**Effect of VEGF-A_165_b on thermal and mechanical hypersensitivity in rats with STZ-induced diabetes.** (A) STZ-induced thermal hypersensitivity was evident at 3 weeks after STZ injection, and was prevented with systemic VEGF-A_165_b [two-way ANOVA, treatment: *F*(2103)=17.26, *P*=<0.0001; time: *F*(3103)=3.74, *P*=0.013; interaction: *F*(6103)=2.59, *P*=0.022. *P<0.05 post-hoc Sidak's test]. (B) STZ-induced mechanical hypersensitivity, measured by a reduction in 50% withdrawal thresholds, was prevented with VEGF-A_165_b by week 3 [two-way ANOVA, treatment: *F*(2104)=6.744, *P*=0.0018; time: *F*(3104)=8.337, *P*<0.0001; interaction: *F*(6104)=3.03, *P*=0.009. *P<0.05, post-hoc Sidak's test]. Arrows indicate STZ injection and the dotted line the start of twice weekly systemic rhVEGF-A_165_b or vehicle treatment. Data shown are mean±s.e.m. (naïve, *n*=11; STZ+PBS, *n*=9; STZ+VEGF-A_165_b, *n*=9). (C) Representative western blots of HMGB1 and actin in hind paw plantar skin at 3 and 7 weeks after STZ injection. (D) HMGB1 protein expression was increased in skin from diabetic rats at both 3 (Kruskal–Wallis statistic 7.367, *P*=0.0132) and (E) 7 weeks [one-way ANOVA *F*(2,12) =15.31, *P*=0.0005]. The change in HMGB1 was not altered with VEGF-A_165_b treatment. Plots show mean±s.e.m. and individual data points [*n*=5 (naïve at both 3 and 7 weeks, STZ 7 weeks), *n*=4 (STZ, 3 weeks). *P<0.05, n.s., not significant, post-hoc Dunn's (3 weeks) and Sidak's tests (7 weeks)].

We induced hyperglycaemia in rats via streptozotocin (STZ) treatment that, with low-dose insulin treatment, was stable and sustained over at least 7 weeks, with little weight loss in the animals (Hulse et al., 2015). Weight loss was not observed in these animals over the 3 weeks of the study (see below). At 3 weeks animals showed significant thermal and mechanical hypersensitivity in the hind paws, which was ameliorated by systemic VEGF-A_165_b treatment (Fig. 1A,B), consistent with published data (Hulse et al., 2015). As previously reported, VEGF-A_165_b treatment had no effect on animal weight or blood glucose levels [weights after 3 weeks of treatment: naïves 289±7 g (mean±s.e.m.), diabetes+vehicle 261±6 g, diabetes+VEGF-A_165_b 264±4 g; blood glucose levels: naïves 6±0.2 mM/l, STZ+vehicle 27±1 nM/l, STZ+VEGF-A_165_b 29±0.8 mM/l; animal weight and glucose levels at 7 weeks were as previously published (Hulse et al., 2015)].

### HMGB1 and RAGE expression is increased in STZ diabetic rats

**Fig. 2.**
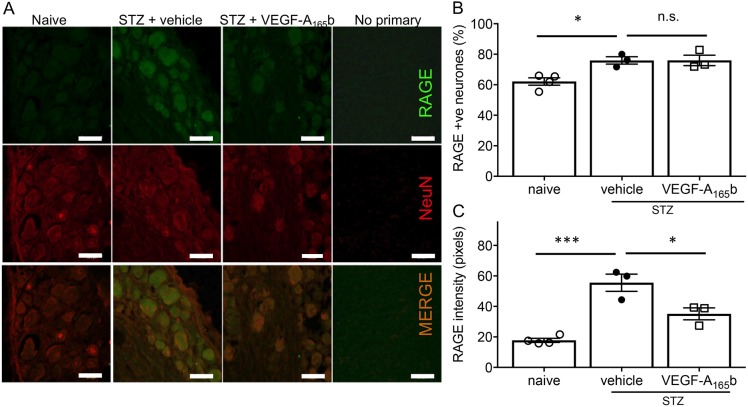
**RAGE is expressed in normal DRG neurons and is increased in rats with STZ-induced diabetes.** (A) Representative images of RAGE (green) and NeuN (neuronal marker, red) staining, showing RAGE expression in DRG neurons in naïve and diabetic rats. (B) The percentage of the total neurons in the DRG expressing RAGE increased in diabetic rats, and this was not affected by VEGF-A_165_b treatment [one-way ANOVA *F*(2,7)=9.104, *P*=0.0113]. (C) RAGE intensity per neuron was increased in diabetic rats, and this increase was partially prevented by VEGF-A_165_b treatment [one-way ANOVA *F*(2,7)=28.37, *P*=0.0004]. Plots show mean±s.e.m. and individual data points; *n*=4 (naïve) and 3 (all STZ groups). *P<0.05; ***P<0.001; n.s., not significant (ANOVA plus post-hoc Sidak's test).

HMGB1 protein expression was significantly increased in hind paw plantar skin at both 3 and 7 weeks post STZ injection. The change in expression of HMGB1 was not affected by treatment with VEGF-A_165_b (Fig. 1C–E). We confirmed RAGE expression in dorsal root ganglia (DRG), which contains the cell bodies of somatosensory neurons (Fig. 2A). RAGE expression was seen in the majority of DRG neurons, across the whole range of cell sizes. RAGE expression is mediated by a positive feedback mechanism upon RAGE activation (Li and Schmidt, 1997), and therefore we determined whether there was any change in RAGE expression in sensory neurons in diabetic rats. The proportion of DRG neurons positive for RAGE expression was increased in diabetic rats compared to naïve rats, as previously reported (Zochodne, 2014), and this was unaffected by VEGF-A_165_b treatment (Fig. 2B). The intensity of the RAGE staining in individual RAGE-positive DRG neurons was also increased in diabetic rats compared to naïve rats, and this increase was partially ameliorated by VEGF-A_165_b (Fig. 2). The changes induced by diabetes were not limited to any particular subgroup of DRG neurons.

### Enhanced TRPV1-evoked Ca^2+^ responses in STZ diabetic rat DRG neurons is a hyperglycaemia-mediated event and is prevented by VEGF-A_165_b treatment

**Fig. 3.**
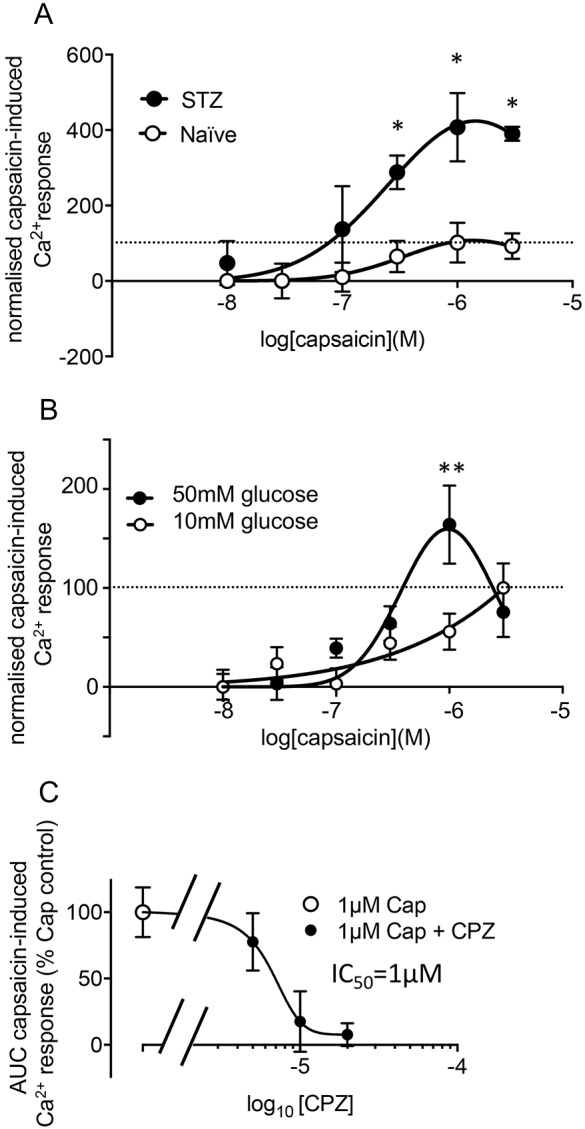
**STZ-induced diabetes *in vivo*, and high-glucose conditions *in vitro*, increase TRPV1 activity in response to agonist in DRG neurons.** (A) Capsaicin-evoked TRPV1 activity was greater in DRG neurons from diabetic rats (*n*=3) at 3 weeks after STZ injection compared to DRG from naïve rats (*n*=4) [two-way ANOVA: interaction *F*(5,28)=2.86, *P*=0.03; capsaicin concentration *F*(5,28)=8.45, *P*<0.0001; STZ/naïve *F*(1,28)=29.19, *P*<0.0001. *P<0.05 post-hoc Sidak's test]. (B) Capsaicin-evoked TRPV1 activity was also enhanced in naïve DRG neurons exposed to 24 h of 50 mM glucose (*n*=5) compared to 10 mM glucose for 24 h [*n*=5, data shown are mean±s.e.m., two-way ANOVA interaction *F*(5149)=2.742, *P*=0.02; glucose concentration *F*(1149)=2.45, *P*=0.12; capsaicin concentration *F*(2, 149)=8.16, *P*<0.0001]. **P<0.01 post-hoc Sidak's test. (C) Capsaicin (Cap)-evoked increase in intracellular Ca^2+^ is blocked by TRPV1 antagonist capsazepine (CPZ) (mean±s.e.m.; *n*=7 replicates for 1 µM Cap; 5 for 1 µM Cap+5 µM CPZ; and 8 for 1 µM Cap plus 10 or 20 µM CPZ). Neurons derived from three rats. Values are normalised to the maximum response evoked in the control conditions for each experiment (indicated by horizontal dashed line in A and B).

TRPV1 contributes to primary sensory neuronal sensitisation in many pain states (Marwaha et al., 2016), including diabetes (Cui et al., 2014; Pabbidi et al., 2008), and the degree of behavioural hypersensitivity is associated with increased agonist-evoked TRPV1 activity. We therefore determined whether DRG neurons isolated from diabetic rats showed increased TRPV1 agonist-evoked Ca^2+^ responses. Capsaicin evoked a larger response in DRG neurons from female diabetic rats compared to DRG neurons from naïve female rats (Fig. 3A). This effect could be modelled in DRG neurons from adult naïve male rats exposed to 24 h of high glucose (50 mM glucose), with an increased intracellular Ca^2+^ fluorescence response to 1 µM capsaicin compared to basal glucose conditions (Fig. 3B). This demonstrates that the enhanced TRPV1-evoked Ca^2+^ responses are, at least in part, a hyperglycaemia-induced effect. The inclusion of equimolar mannitol in the basal glucose condition indicates that effects of 50 mM glucose cannot be attributed to any osmotic effect of the additional glucose. The specific TRPV1 antagonist capsazepine blocked the capsaicin-evoked change in intracellular Ca^2+^ in primary sensory neurons in this assay in a concentration-dependent manner with an IC_50_ of ∼1 µM (Fig. 3C).

**Fig. 4.**
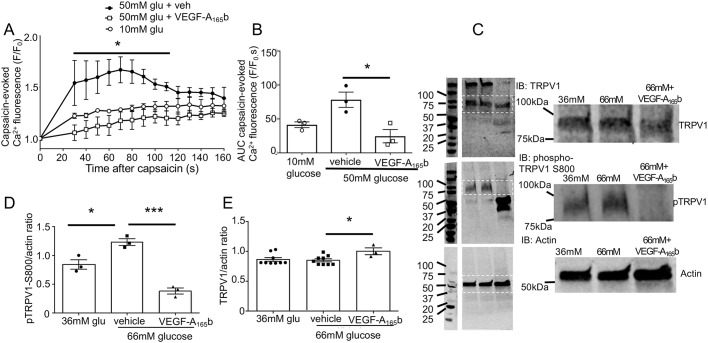
**VEGF-A_165_b blocks high-glucose-mediated TRPV1 sensitisation and phosphorylation in DRG neurons.** (A) Capsaicin-evoked TRPV1 activity was enhanced in primary DRG neurons cultured in 50 mM glucose, and this was prevented with VEGF-A_165_b co-treatment [*n*=3 per treatment, two-way ANOVA interaction *F*(28,90)=1.081 (ns, not significant), treatment *F*(2,90)=67.05, *P*<0.0001, time *F*(14, 90)=3.842, *P*<0.0001. *P<0.05 vehicle compared with VEGF-A_165_b, post-hoc Tukey's test]. (B) Total capsaicin-evoked activity (AUC) was increased in high-glucose conditions and reduced by VEGF-A_165_b [one-way ANOVA *F*(2,6)=9.424, *P*=0.014, *P<0.05 post-hoc Sidak's test]. (C) Representative western blots (IB) for TRPV1 (96 kDa), pS800 TRPV1 (95 kDa) and actin (42 kDa) in 50B11 DRG neurons treated for 24 h in basal glucose conditions (36 mM+30 mM mannitol), high-glucose conditions (66 mM) with or without VEGF-A_165_b treatment (intervening lanes between markers and samples removed for clarity). A magnification of the boxed area is shown on the right. (D) Phosphorylated TRPV1 protein levels in 50B11 neurons increased in high-glucose conditions compared to basal glucose conditions, and this was prevented with VEGF-A_165_b treatment [*n*=3 per treatment; one-way ANOVA *F*(2,6)=42.01, *P*<0.0003. *P<0.05, ***P<0.001 post hoc Sidak's test]. (E) TRPV1 protein levels in 50B11 neurons were slightly increased in high glucose plus VEGF-A_165_b conditions [one-way ANOVA *F*(2,18)=3.637, *P*=0.047, *n*=9 in 36 mM glucose and 66 mM glucose+ vehicle, *n*=3 for 66 mM glucose+VEGF-A_165_b as shown, *P<0.05 post-hoc Sidak's test]. Data shown are mean±s.e.m., plus individual data points in B, D and E. (Please see methods text for different glucose concentrations in 50B11 and primary DRG neuronal cultures).

The high-glucose-mediated increased TRPV1-evoked Ca^2+^ responses were significantly reduced when DRG neurons were co-treated with VEGF-A_165_b in high-glucose conditions (Fig. 4). TRPV1 sensitisation, which leads to increased Ca^2+^ influx, has previously been attributed to increased phosphorylation, particularly at S800 (Mandadi et al., 2006; Wang et al., 2015), which is a protein kinase C (PKC)-dependent phosphorylation site. In immortalised embryonic rodent DRG neurons (50B11) exposed to high-glucose conditions, phosphorylation of TRPV1 at S800 increases compared to that seen in basal glucose conditions, with no effect on total TRPV1 expression (Radu et al., 2013, see also Khomula et al., 2013; Mohammadi-Farani et al., 2014). Both high-glucose-enhanced TRPV1 Ca^2+^ responses (Fig. 4A,B) and TRPV1 phosphorylation (Fig. 4C,D) were reduced after 24 h exposure to VEGF-A_165_b, even though TRPV1 expression was slightly increased by VEGF-A_165_b treatment (Fig. 4E).

### Increased RAGE activity contributes to high-glucose-induced TRPV1-evoked Ca^2+^ responses in DRG neurons

**Fig. 5.**
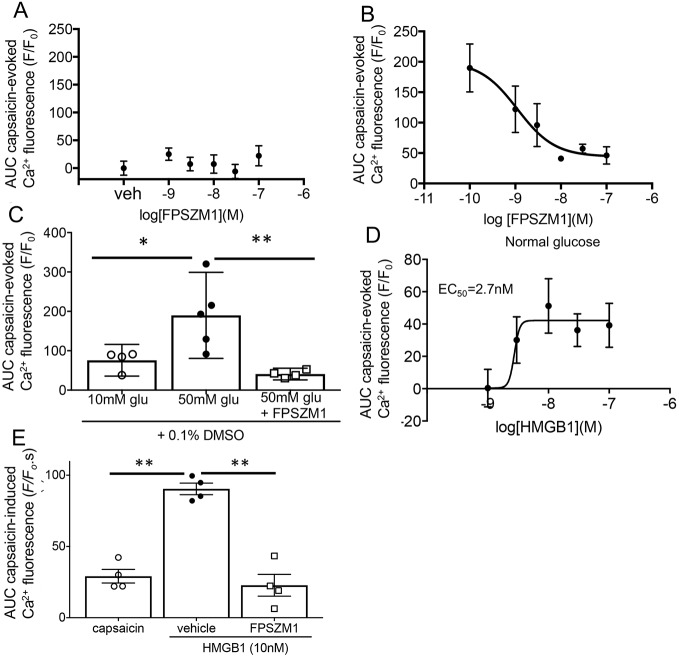
**RAGE agonism and antagonism modulate capsaicin-evoked intracellular Ca^2+^ changes in DRG neurons.** (A) FPSZM1 had no effect on capsaicin-evoked TRPV1 activity at any concentration in 10 mM glucose conditions (*n*=4 per treatment). (B) FPSZM1 significantly reduced the capsaicin-evoked TRPV1 activity in 50 mM glucose conditions in a concentration-dependent manner (*n*=4 per treatment). (C) The high-glucose-induced increase in TRPV1 activity was completely prevented when neurons were co-treated with 10 nM FPSZM1 [*n*=4, 10 mM glucose and 50 mM glucose+FPSZM1; *n*=5, 50 mM glucose; one way ANOVA *F*(2,10)=8.398, *P*=0.0072. *P<0.05, **P<0.01 post-hoc Sidak's test]. (D) HMGB1 increased the capsaicin-evoked TRPV1 activity in a concentration-dependent manner [one-way ANOVA *F*(95,30)=2.688, *P*=0.04. *P<0.05 post-hoc Bonferroni tests compared with control, *n*=6 per treatment]. (E) The HMGB1-mediated (10 nM) increase in capsaicin-evoked TRPV1 activity was blocked by 10 nM FPSZM1 [*n*=4 per treatment, one-way ANOVA *F*(2,9)=42.63, *P*<0.0001]. Data are mean±s.e.m. and individual data points as shown. *P<0.05, **P<0.01 post-hoc Sidak's tests.

As the level of the RAGE agonist HMGB1 was increased in the skin (i.e. in the tissues surrounding nociceptive neuronal terminals) of diabetic animals, we hypothesised that the altered neuronal sensitivity to TRPV1 agonists seen *in vitro* could be RAGE dependent. The RAGE antagonist FPSZM1 did not affect capsaicin-evoked TRPV1 Ca^2+^ changes in normal glucose concentrations (Fig. 5A), but reduced Ca^2+^ fluorescence in high-glucose conditions in a concentration-dependent manner (Fig. 5B). Concentrations of FPSZM1 above 10 nM reduced capsaicin-evoked Ca^2+^ responses to control levels (Fig. 5C).

### HMGB1 increases TRPV1-agonist-evoked Ca^2+^ responses, which is a RAGE-mediated effect that is blocked with VEGF-A_165_b

**Fig. 6.**
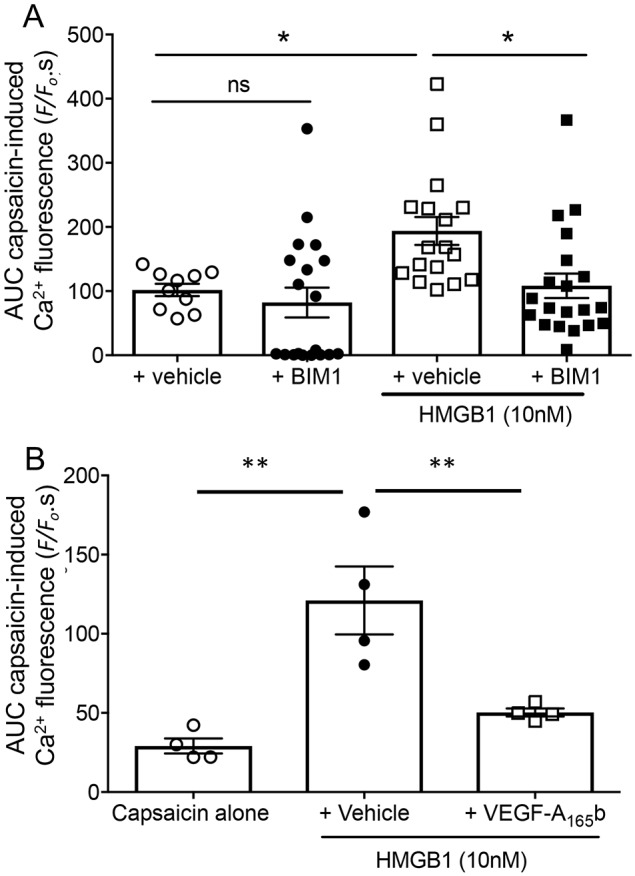
**HMGB1-mediated sensitisation of capsaicin-evoked TRPV1 activity is PKC-dependent.** (A) BIM1 had no significant effect on capsaicin-evoked Ca^2+^ response alone, but significantly reduced HMGB1-evoked responses (one-way ANOVA *F*=5.627, *DF*=3, *P*=0.0018. *P<0.05; ns, not significant compared with HMGB1+BIM1 and control post-hoc Bonferroni tests). Capsaicin+vehicle, *n*=10; capsaicin+BIM1, *n*=19; capsaicin+HMGB1+vehicle, *n*=17; capsaicin+HMGB1+BIM1, *n*=20 as shown. (B) VEGF-A_165_b significantly reduced HMGB1-mediated sensitisation of capsaicin-evoked TRPV1 activity [one-way ANOVA *F*(2,9)=14.2, *P*=0.0016. **P<0.01 post-hoc Sidak's test]. Data shown are mean±s.e.m., *n*=4 per group.

HMGB1 is a RAGE agonist that has been implicated in peripheral nociceptive signalling (Feldman et al., 2012). HMGB1 increased capsaicin-evoked TRPV1 Ca^2+^ responses in DRG neurons (10 mM glucose; Fig. 5D) and this was reduced by incubation with 10 nM FPSZM1 (Fig. 5E). Inhibition of PKC activity by incubation of DRG neurons with HMGB1 and 1 µM BIM-1 reduced capsaicin-evoked TRPV1 Ca^2+^ changes (Fig. 6A). HMGB1-enhanced TRPV1-evoked Ca^2+^ responses were also reduced following 24 h treatment with VEGF-A_165_b (Fig. 6B).

Effect sizes for data in all experiments are shown in Table S1.

## DISCUSSION

These data collectively show for the first time that, *in vitro*, (1) high glucose concentrations alter TRPV1-evoked Ca^2+^ responses in DRG neurons through RAGE, (2) HMGB1 alters TRPV1-evoked Ca^2+^ responses through RAGE in DRG neurons under basal glucose conditions, in a manner that is PKC dependent and blocked by VEGF-A_165_b, and (3) HMGB1 is upregulated in the skin in diabetic rats. This is the first study to demonstrate a potential neuronal mechanism through which HMBG-1–RAGE signalling could alter DRG neuronal properties under high-glucose conditions *in vitro*.

There have been many proposed causes for painful diabetic neuropathy. These include microvasculopathic neuronal hypoxia, direct effects of glucose toxicity on sensory neurons, and activation of TRPA1 and other ion channels in sensory neurons by reactive metabolites, such as methylglyoxal (Andersson et al., 2013; Huang et al., 2016). In the later stages of the condition, RAGE activation by AGEs is driven by molecules such as methylglyoxal, resulting in cellular damage and further complications (Hidmark et al., 2014). RAGE is a pattern recognition receptor that is capable of signalling local tissue damage (Kato et al., 2016). Activation of RAGE by AGEs usually results after significant accumulation of AGEs over time (months to years *in vivo*), and is considered to be a late contributor to diabetic complications (Singh et al., 2014). Herein, we show that neuronal RAGE activity in sensory neurons is not detectable in neurons cultured in normal glucose concentrations, but is evident following a short (24 h) exposure to high glucose (50 mM) *in vitro*. AGE formation is reported to occur after 6–10 days in 2 M glucose *in vitro* (Hori et al., 2012), suggesting that our experimental conditions would not be sufficient to cause AGE generation. Thus, we hypothesise that neuronal RAGE activation after a short exposure to high levels of glucose, possibly through the more rapid generation of HMGB1, could contribute to early changes in neuronal properties, resulting in enhanced nociception.

Importantly, the endogenous RAGE agonist HMGB1 itself alters TRPV1-evoked neuronal responses under normal glucose conditions. HMBG1 has been found in Schwann cells, DRG satellite cells and primary sensory neurons during traumatic neuropathy (Wan et al., 2016), in sensory neurons during experimentally induced diabetes (Juranek et al., 2013) and in the human diabetic nerve (Bierhaus et al., 2004). HMGB1 has been implicated in peripheral mechanisms of pain through both direct (Allette et al., 2014) and indirect activation of sensory neurons through interaction with pro-inflammatory molecules such as interleukin (IL)-1 and TNF (Wan et al., 2016), and RAGE-mediated pro-inflammatory cytokine release from immune cells (Kato et al., 2016). Taken together, our data support a direct effect of HMGB1 on sensory neurons, with rapid (≤24 h) onset, that can be induced under high-glucose conditions *in vitro*, extending our understanding of the function of HMGB1 and its effects on functional neuronal change. As the RAGE agonist HMGB1 is increased in skin in diabetic rats when pain behaviour is altered (data herein, and Hulse et al., 2015), and RAGE expression is increased in sensory neurons (Zochodne, 2014), these peripheral actions of HMGB1 may be a contributory factor in the pain associated with glucose-induced neuronal damage and pain in diabetes.

Modulation of the TRP channels TRPA1 and TRPV1 has been implicated in both neuronal damage and pain in diabetic neuropathy (Cui et al., 2014; Hong and Wiley, 2005; Khomula et al., 2013). TRPA1 and TRPV1 are important molecules in the sensitisation of peripheral nociceptive afferents (Cheng and Ji, 2008), and TRPV1 is directly sensitised by high-glucose conditions in sensory neurons (Lam et al., 2018) and during diabetes *in vivo* (Hong and Wiley, 2005; Khomula et al., 2013). TRPV1 phosphorylation by PKC at S800 is key to its sensitisation (Wang et al., 2015). Both S800-TRPV1 phosphorylation and sensitisation can be increased in high-glucose conditions *in vitro*, and after RAGE activation by HMGB1. TRPV1 is also phosphorylated and sensitised through a PKC-dependent mechanism during diabetes *in vivo* (Hong and Wiley, 2005). RAGE–PKC interactions can also modulate neuronal function *in vivo*, affecting conduction velocity slowing (indicative of peripheral neuronal damage), as well as neuronal repair in diabetic neuropathy (Zochodne, 2014). While other signalling pathways, such as those mediated by PKA, are also known to contribute to sensory neuronal sensitisation (Cheng and Ji, 2008), our data are consistent with PKC-mediated phosphorylation of sensory neuronal TRPV1 being one important mechanism through which HMGB1, and presumably other RAGE agonists, could rapidly sensitise peripheral neurons. This could contribute to pain in conditions where HMGB1 is expressed, such as various neuropathic conditions (Allette et al., 2014; Brederson et al., 2016; Juranek et al., 2013).

While TRPV1 expression changes have been reported in specific DRG neuronal subpopulations, total TRPV1 levels were reported to decrease in DRG neurons in diabetic rats (Hong and Wiley, 2005). Despite this overall decrease in TRPV1 expression, neuronal TRPV1 functional responses were increased (sensitised) through a PKC-dependent mechanism (Hong and Wiley, 2005). This agrees with our findings under high-glucose conditions *in vitro* and suggests that our reported differences between naïve and diabetic DRG Ca^2+^ responses *ex vivo* are not attributable to alteration in TRPV1 expression levels alone. We found no change in neuronal TRPV1 expression in high-glucose conditions (Fig. 4). It should also be noted that in measuring intracellular Ca^2+^ levels, albeit evoked by a TRPV1 agonist, the enhanced responses we report might represent TRPV1 sensitisation *in vitro* and *ex vivo*, but could also be attributable to changes in other ion channels. RAGE can directly contribute to PKC-dependent capsaicin-induced non-TRPV1-mediated intracellular Ca^2+^ increases in high-glucose conditions (Lam et al., 2018). TRPA1 is also sensitised in rat DRG immortalised neurons (50B11) in high-glucose conditions *in vitro* (Hulse et al., 2015) and in animal models of diabetes. TRPA1 forms complexes with TRPV1, and can affect TRPV1 function, such as reducing desensitisation, thus potentially altering Ca^2+^ responses (Masuoka et al., 2017; Staruschenko et al., 2010). There is, however, no evidence that RAGE can directly alter TRPA1 function. Voltage-gated Na^+^ and Ca^2+^ channels are also involved in diabetic pain in STZ rodents, for example, through methylglyoxal-RAGE-mediated effects on Nav1.8 (Huang et al., 2016), TNF/NFκB-mediated upregulation of Nav1.7 (Huang et al., 2014) or upregulation of Ca^2+^ channel subunits (Yusaf et al., 2001). All of these effects will contribute to the known changes in nociceptor properties in diabetes *in vivo* (Chen and Levine, 2001), and could also contribute to the *in vitro* changes that we report.

Hypoxia, which is known to affect peripheral tissues including nerves, is a potent stimulus for TRPV1 sensitisation through hypoxia-inducible factor (HIF)-1α and PKC (Ristoiu et al., 2011). Hypoxia and HIF-1α also induce expression of the pro-nociceptive VEGF-A isoform VEGF-A_165_a, which also sensitises TRPV1 through PKC (Hulse et al., 2014). We have previously shown that twice weekly treatment with recombinant human (rh)VEGF-A_165_b reverses both pain and neuronal terminal loss during experimental diabetes, and sensitisation of sensory neuronal TRPA1-evoked responses (Hulse et al., 2015). Given that RAGE expression is upregulated by HMGB1, the reduction in RAGE intensity *in vivo* upon VEGF-A_165_b treatment (Fig. 3C) suggests that this treatment could reduce HMGB1 activation of RAGE or modify the downstream signalling from HMGB1–RAGE activation that results in the feed-forward agonist-induced upregulation of RAGE, resulting in reduced pain, rather than exerting direct effects on neuronal TRPV1. This requires confirmation, as does the contribution of HMGB1–RAGE–TRPV1 to altered pain behaviour in diabetic neuropathy *in vivo*. Blockade of HMGB1–RAGE-mediated neuronal sensitisation by rhVEGF-A_165_b *in vitro* supports our hypothesis that alteration of VEGF-A isoforms, for example by treatment with rhVEGF-A_165_b or an agent that alters VEGF-A splicing to favour VEGF-A_165_b may be an effective treatment for diabetic neuropathy.

## MATERIALS AND METHODS

Cell culture reagents (media and media supplements) and equipment were supplied by Invitrogen Life Technologies (Paisley, UK). Protein extraction buffer, supplements, buffers and solutions, streptozotocin and sodium pentobarbital were supplied by Sigma-Aldrich (Irvine, UK) and western blotting reagents and equipment through Bio-Rad (CA, USA). Superfrost plus slides and OCT embedding medium were supplied by VWR International (PA, USA) and DAPI by Vector Laboratories, (Peterborough, UK). Behavioural testing equipment was supplied by Ugo Basile (Varese Italy).

Drugs were supplied by R&D Systems (MA, USA) (rhVEGF-A_165_b and rhHMGB1), EMD Millipore (MA, USA) (FPSZM1) and LinShin (insulin pellets). Details of antibodies and sources are shown in Table S2.

A total of 63 male and female rats (Charles River Laboratories, Kent, UK) were used in this study. All experiments were carried out in laboratories at the University of Nottingham in accordance with the EU 2010/63 Directive, the United Kingdom's Scientific Procedures Act 1986 and associated amendments and guidelines (2012) and were approved by the University of Nottingham AWERB (Animal Welfare and Ethical Review Board). The experiments in these studies conformed to the principles of the ARRIVE guidelines. Animals were housed at 21°C and 55% relative humidity with a 12-h-light–12-h-dark cycle. For diabetic animals, food and water were provided *ad libitum* except immediately prior to STZ injection.

Humane endpoints included loss of body weight of >15% or animal behaviour indicative of pain or distress, such as staring coat, immobility or hunched stance (no animals met any of these endpoints). All rats were group housed (minimum of two animals per cage) under 12-h-light–12-h-dark cycles, with additional paper bedding material and environmental enrichment, and *ad libitum* access to standard chow and water. Owing to increased urination in diabetic animals, bedding and cage materials were changed frequently, usually at need rather than at specific time intervals.

Adult female Sprague Dawley rats (∼250 g, *n*=35) were used in the *in vivo* diabetes study. Adult female Sprague Dawley rats were used for STZ-induced diabetes model as these animals reach the top of their growth curve at about this age. It is therefore easier to monitor animal welfare, as even small amounts of weight loss in comparison to untreated control animals is evident with correction for any growth-related weight gain (Calcutt, 2004). Adult male Wistar rats (∼250–350 g, *n*=29) were used for primary DRG neuronal cultures *in vitro*, as previously reported (Hulse et al., 2014, 2015).

Animal numbers were determined *a priori* based on effect sizes in multiple measures derived from a previous group of animals that underwent the same procedures in a previous study using G*Power software (Faul et al., 2007; Hulse et al., 2015).

Diabetes was induced with an intraperitoneal injection (i.p.) of streptozotocin (STZ; 50 mg per kg body weight) as previously described (Hulse et al., 2015). Hyperglycaemia (>15 mM circulating glucose) was confirmed 4 days after STZ injection; in animals in which hyperglycaemia was not confirmed after initial STZ injection, a second STZ injection was administered. Animals in which STZ injection did not result in hyperglycaemia (*n*=6) were not used in further experiments. STZ-injected hyperglycaemic animals were treated with very low-dose insulin supplementation with one-third of a LinShin insulin pellet injected subcutaneously in the scruff of the neck under brief general isofluorane anaesthesia (2-3% in O_2_), and using the sterile trocar supplied by the manufacturer (Calcutt, 2004; Hulse et al., 2015). Animals were supplied with concentrated sucrose solution to reduce the incidence of hypoglycaemia in the 24 h immediately post-STZ injection (Calcutt, 2004). Experimental groups were: diabetic rats treated twice weekly with recombinant human (rh)VEGF-A_165_b injection (20 ng per g body weight, *n*=9) or phosphate-buffered saline (PBS, VEGF vehicle, *n*=9) (Hulse et al., 2015). Age-matched naïve rats (*n*=11) served as non-diabetic controls. Twice weekly injections of VEGF-A_165_b and PBS began 1 week after confirmation of both hyperglycaemia and altered nociceptive behaviour, a treatment regime we have previously shown to be effective in traumatic and diabetic neuropathies (Hulse et al., 2014, 2015). Animals were maintained for a further 3 weeks after confirmation of hyperglycaemia. Weekly thermal and mechanical nociceptive testing was performed throughout the duration of the study.

Rats were habituated to nociceptive behavioural testing environments for 2 weeks prior to STZ injection and for 20 min before the start of each weekly session. Thermal nociceptive testing was performed using the Hargreaves test (Hargreaves et al., 1988). The intensity of the radiant heat source was set so that the mean response latency was ∼10 s in naïve animals at baseline testing, and the intensity was kept constant throughout the duration of the study. Three latencies were measured in each testing session and the mean value calculated. To prevent heat-induced tissue damage, an inter-stimulus interval of a minimum of 5 min was used between measurements. Mechanical nociceptive testing was performed using von (v)Frey monofilaments as previously described (Hulse et al., 2014, 2015). vFrey monofilaments of varying force were applied to the plantar surface of the hind paw five times each to generate a stimulus–response curve and the withdrawal threshold (force in grams) was calculated as the force at which the animal withdrew to 50% of stimulus presentations. For both thermal and mechanical testing, measurements were taken for both left and right hind paws and the data were treated as duplicates for each animal. The animal was taken as the independent observation, not the paw. The operator performing behavioural testing was blinded to the treatment groups, as another researcher coded the injections for treatments. As the operator administering the injections was blinded to the treatment, this resulted in a randomisation of treatment order.

After 3 weeks, rats were terminally anaesthetised (with sodium pentobarbital at 60 mg per kg body weight, i.p.) and transcardially perfused with PBS. Plantar skin and L3/4/5 DRG neurons were dissected and (1) immediately snap-frozen on dry ice and then transferred to −80°C before processing for protein extraction or (2) DRG neurons were dissected and post-fixed for 24 h in 4% paraformaldehyde (PFA, pH 7.4) at 4°C and then transferred to 30% sucrose solution for a further 24 h at 4°C for immunofluorescence studies.

### *In vitro* assessment of TRPV1 expression and phosphorylation

50B11 neurons (a gift from Ahmed Hoke, provided through Damon Lowes, see Acknowledgements) are an immortalised DRG sensory neuronal cell line derived from embryonic rats (Chen et al., 2007) and were used as a model system to determine changes in total TRPV1 and phosphorylated TRPV1 under different glucose conditions, and to reduce the numbers of animal numbers used. These cells were authenticated in-house as expressing functional neuronal markers, particularly those associated with nociception such as TRPA1 and TRPV1 (Bestall, 2017). 50B11s were maintained in neurobasal medium supplemented with 10% fetal bovine serum, 0.55 mM glutamine, B27 supplement and an additional 11 mM glucose (making the total glucose concentration 36 mM; Chen et al., 2007). Basal glucose concentration is thus higher for 50B11 maintenance than for primary sensory neurons, but is required for their culture. 50B11 neurons have impaired neurite outgrowth in both normal (5 mM) and high (66 mM) glucose conditions [S. M. Bestall, ‘Diabetic neuropathy: A mechanism of TRPV1 sensitisation and the treatment with Vascular Endothelial Growth Factor-A165b (VEGF-A165b)’, PhD thesis, University of Nottingham, 2017].

50B11 neurons were differentiated for 24 h with 1 nM nerve growth factor (NGF) and 75 µM forskolin, and then treated for a further 24 h under the following conditions: basal glucose (36 mM glucose+30 mM mannitol), and high glucose (66 mM glucose) with or without recombinant human VEGF-A_165_b (2.5 nM) or PBS (VEGF vehicle). After 24 h, protein was extracted and subject to western blotting for TRPV1 and TRPV1 phosphorylated on S800 (p800-TRPV1). Equimolar mannitol addition controlled for any potential effect of increased osmolarity under high-glucose conditions.

### Western blotting

Protein was extracted in RIPA buffer containing 150 mM sodium chloride, 1% nonyl phenoxypolyethoxylethanol (NP-40), 0.5% sodium deoxycholate, 0.1% SDS and 50 mM Tris-HCl pH 8.0 supplemented with protease inhibitor cocktail (20 µl ml^−1^ buffer; 1 mM phenylmethylsulfonyl fluoride, 10 mM sodium orthovanadate and 50 mM sodium fluoride). Equal amounts of protein were loaded and separated on a 4–20% gradient precast SDS-PAGE gel and proteins were transferred to a PVDF membrane using wet transfer. Membranes were incubated in blocking buffer (5% bovine serum albumin in Tris-buffered saline containing 0.1% Tween 20) for 60 min at room temperature and incubated overnight at 4°C in primary antibodies against TRPV1, p800-TRPV1 and HMGB1 (Table S2) diluted in blocking solution. Loading was confirmed and differences determined by concurrent probing for actin expression. Membranes were then washed in PBS and fluorescent secondary antibodies (Table S2) were applied in blocking solution for 60 min at room temperature. Membrane fluorescence was visualised and analysed on a Licor Odyssey Fc.

### Immunofluorescence

Following fixation and cryoprotection of tissue from diabetic rats, DRG were embedded and frozen at −80°C before sectioning at 8 µm thickness. Sections were then stored mounted on slides (Superfrost plus) at −80°C. Sections were washed in PBS, blocked with 5% bovine serum albumin and 10% fetal bovine serum in PBS containing 0.2% Triton X-100. Sections were incubated in primary antibodies against RAGE and NeuN (Table S2) in blocking solution overnight at 4°C. Slides were washed in PBS and incubated in fluorescent secondary antibodies in PBS containing 0.2% Triton X-100 for 2 h at room temperature, then cover slips were added with Vectorshield mounting medium containing DAPI (4′,6-diamidino-2-phenylindole). A minimum of five randomly selected non-serial images were acquired from at least five sections from each animal per treatment group at 20× magnification using consistent microscope settings between channels on a Leica TCS SPE confocal microscope and the Leica applications suite software (LAS X). All immunofluorescence images were equally enhanced (30% increase in brightness, 0% change in contrast) for presentation (Fig. 3). Images were exported for further analysis in Image J software (specifically FIJI) (Schindelin et al., 2012). The number of RAGE-positive neurons was determined by counting the number of NeuN-positive DRG neurons that co-expressed RAGE. RAGE intensity analysis was performed using the mean grey area values for these neurons. For each image containing RAGE-positive neurons, a local mean grey area background value was subtracted from experimental values. All image analysis was performed on raw images by an experimenter who was blind to treatment group. The images shown in Fig. 2 are altered for brightness (+35%) and contrast (−20%) compared to the raw images used for analysis. The RAGE antibody (Table S2) detected a single band of ∼45 kDa, as expected, in 50B11 neurons (Fig. S1).

### TRPV1 activity *in vitro*

For primary sensory neuronal culture, which is widely used to study mechanisms of neuronal signalling and sensitisation using single-cell approaches (Huang et al., 2015) through to high-throughput screening (Hulse et al., 2014; Newberry et al., 2016), adult male Wistar rats were killed by anaesthetic overdose (60 mg kg^−1^ sodium pentobarbital, i.p. injection) and death verified by confirmation of cessation of the circulation.

T1–L6 DRGs were dissected, enzymatically and mechanically dissociated, and cultured on poly-L-lysine- and laminin-coated 96-well plates (black sided, Costar) at a seeding density of 2000 cells per well in Ham's F12 medium containing 1× N2 supplement, 1% BSA and 1% penicillin-streptomycin. Equal numbers of cells were seeded per well to reduce the inter- and intra-assay variability between wells. After cell attachment, cultures were treated with 30 µg ml^−1^ 5-fluoro-2′-deoxyuridine to prevent mitosis of non-neuronal cells. Neuronal cultures were maintained in basal glucose conditions (10 mM glucose with 40 mM mannitol as an osmotic control) or high-glucose conditions (50 mM glucose) for 24 h with or without the following co-treatments: 2.5 nM rhVEGF-A_165_b, rhHMGB1 (1–100 nM), FPSZM1 (RAGE antagonist 1–100 nM) and 1 µM BIM-1 (PKC inhibitor). Cells were then loaded with Fluo-4 (Invitrogen) in Hank's balanced buffered saline solution containing 20 mM HEPES and 2 mM CaCl_2_ for 1 h. The TRPV1 agonist capsaicin [1 µM unless otherwise stated, concentration chosen based on previous findings (Hulse et al., 2014) known to be in the range for agonist-specific TRPV1-evoked responses (Edwards, 2014)] was used to stimulate TRPV1 channels and the resulting changes in intracellular Ca^2+^ fluorescence were measured on a Victor X4 plate reader at 37°C over a 160 s period. In some experiments, to verify the Ca^2+^ response as being evoked through activation of TRPV1, primary neuronal cultures were dissociated, plated and maintained in basal glucose for 24 h. Following loading with Fluo-4, capsazepine or vehicle (0.03% DMSO) was added to the culture 20 min prior to addition of 1 µM capsaicin and the Ca^2+^ assay. In all assays, the baseline readings were determined prior to initial capsaicin application and under control conditions to determine any background signal. The sequential fluorescence recordings were corrected for background, and expressed as a fold change of respective baseline values to reduce inter-well variance and to control for minor variations in cell number (Hulse et al., 2014). The inter-assay mean coefficient of variation was 8.9% (from *n*=11 independent experiments, each containing 3–20 repeats all performed by S.M.B.). Fluorometric imaging plate assays such as this are widely used to assay TRPV1 responses to capsaicin in transfected and native cells, and responses are known to be TRPV1 dependent (Gunthorpe et al., 2007; Pineau et al., 1996; Rami et al., 2006; Tafesse et al., 2004; Wang et al., 2014; Zhuang et al., 2004). Each plate contained DRG neurons from a single rat and in each replicate plate, cells were exposed to the same combination of experimental treatment conditions in a block design, to allow comparison of responses within and between plates. For reproducibility, each assay was performed multiple times as noted in the text, and each independent plate was exposed to identical treatments in each experiment. Ca^2+^ levels were normalised to baseline measures in each plate to allow for pooling of data derived from replicates, as is usual with repeated high-throughput assays (Iversen et al., 2004).

This assay uses TRPV1 as a read-out for altered nociceptive neuronal activity, and thus these experiments were not designed to investigate precise mechanisms of TRPV1 modulation. We have previously validated this high-throughput assay for its ability to replicate the effects of compounds assayed by single-cell patch clamp analysis (Hulse et al., 2014). The use and comparison of capsaicin-evoked Ca^2+^ responses in each assay limits the comparisons being made to the responses of the TRPV1-expressing nociceptive population of sensory neurons in the rat, ensuring comparison of like responses between assays. The TRPV1-expressing population represents ∼65% of DRG nociceptors (peptidergic plus IB4^+^ populations; Price and Flores, 2007).

### Experimental design, data extraction and analysis

Effect sizes and representative data values for experiments in this manuscript are given in Table S1. TRPV1 activity assay sample size calculations were determined using previously published data (Hulse et al., 2014, 2015) and the following parameters: repeated measures ANOVA, between factor, power 0.8, number of groups as 3, and number of measurements per group as 21. Sample size calculation for behavioural experiments was based on a two-tailed non-parametric analysis (Mann–Whitney), with a minimum required power of 0.8. Numbers of animals used were calculated to give sufficient power for further tissue analyses on subgroups given the possibility for tissue loss and small tissue samples (DRG).

All data are presented as mean±s.e.m. and *n* values given represent independent observations, which are usually individual animals or independent cultures/treatments. Multiple group comparisons were made by one-way ANOVA (treatment effects), Kruskal–Wallis (KW, treatment effects) or two-way ANOVA (treatment effects over time, or with agonist concentration), all two-tailed tests, as stated in the figure legends. Overall effects of different condition effects on neuronal Ca^2+^ over time were compared by comparison of integrated area under the curve (AUC) for each independent assay followed by comparison using parametric/non-parametric ANOVA as stated in the figure legends. For concentration–response curves (Figs 4 and 6), each individual data point comprises the mean AUC, rather than peak Ca^2+^ response, from the stated number of experimental repeats. Pair-wise post-hoc comparisons were made using Sidak's tests or Dunn's tests as stated in figure legends, with correction for multiple comparisons, when ANOVA significance was reached. Alpha was set at 0.05. All statistical analysis was performed using GraphPad Prism version 6–7 for Windows and Mac, (GraphPad Software, La Jolla, CA; www.graphpad.com).

## Supplementary Material

Supplementary information
