# Supplementary material for: Sensory neuronal sensitisation occurs through HMGB-1–RAGE and TRPV1 in high-glucose conditions
Source: J Cell Sci. 2018 Jul 26;131(14):jcs215939. doi: 10.1242/jcs.215939 (PMC6080605; doi:10.1242/jcs.215939)
Supplement: Supplementary information [file joces-131-215939-s1.pdf]

Table S1. Representative data and effect sizes for outcome measures shown in the text.

Effect sizes calculated using G*Power (Faul et al., 2007).

Parameter<br>Diabetic rat experiments	Naïve/control Mean±SD (n)	Diabetic Mean±SD(n)	Diabetic+VEGF-A$_{165}$b Mean±SD (n)	Effect size (n)<br>(Cohen's d)	Figure
Behaviour - thermal	12.9 ± 2.9 (11)	7.9 ± 1.2 (9)	11.7 ±2.1 (9)	1.05 (28)	1
Behaviour – von Frey	13.4 ±7.2 (11)	4.5 ± 3.2 (9)	9.5 ± 4.3 (9)	0.74 (29)	1
HMGB1 western blots	0.96±0.7 (5)	5.4 ± 3.6 (4)	3.7 ± 2.1 (4)	0.94 (13)	2B
RAGE expression-%	62 ± 4.8 (4)	76 ± 4.1 (3)	76 ± 5.9 (3)	1.37 (11)	3B
RAGE expression-intensity	17.8 ± 2.7 (4)	55.6 ± 9.7 (3)	35.2 ± 6.7 (3)	2.6 (11)	3C

In vitro DRG/50B11 experiments	Control	Intervention (high glucose/ HMGB1)	Intervention + VEGF-A$_{165}$b/FPSZM1	Effect size (n)	Figure
TRPV1 activity<br>(DRG neurons)	41.4 ± 7.3 (3)	78.3 ± 19.5 (3)	24.6 ± 17 (3)	1.32 (9)	5B
TRPV1<br>pTRPV1 (50B11 neurons)	0.86 ± 0.09 (9)<br>0.84 ± 0.14 (3)	0.85 ± 0.07 (9)<br>1.23 ±0.1 (3)	1.002 ± 0.09 (3)<br>0.38 ± 0.09 (3)	0.56 (21)<br>3.48 (9)	5E<br>5D
TRPV1 activity (+HMGB1)	29 ± 9.6 (4)	121 ± 43 (4)	50 ± 5 (4)	3.93 (12)	8B
TRPV1 activity<br>(high glu ± FPSZM1)	75.8 ± 25 (4)	189.9 ± 88 (5)	40.9 ± 9.3 (4)	2.42 (13)	6C
TRPV1 activity<br>(HMGB ± FPSZM1)	29.1±4.8 (4)	90.4±4.1 (4)	22.8±7.7 (4)	2.65 (12)	7B

Faul, F., Erdfelder, E., Lang, A. G. and Buchner, A. (2007). G*Power 3: a flexible statistical power analysis program for the social, behavioral, and biomedical sciences. *Behav Res Methods* 39, 175-91.

Table S2. Antibody information

Target	Antibody type	Source and catalogue number	Dilution	Ref
Primary antibodies–Western blotting				
TRPV1	Rabbit polyclonal	Abcam (Ab10296)	1:500	(Hulse et al., 2014)
p800-TRPV1	Rabbit polyclonal	Abnova (PAB8499)	1:250	(Mandadi et al., 2006)
HMGB1	Rabbit polyclonal	Abcam (Ab18256)	1:1000	(Xiong et al., 2016)
Actin	Goat polyclonal	Santa Cruz (sc-1615)	1:200	(Stanojlović et al., 2016)
Primary antibodies–immunofluorescence				
RAGE	Rabbit polyclonal	AbCam (Ab37647)	1:500	(Shirley et al., 2014)
NeuN	Mouse monoclonal	Millipore (MAB377)	1:200	(Krzisch et al., 2015)
Secondary antibodies–Western blotting				
Anti-rabbit	Donkey	Licor IRDye 680RD (926-68071)	1:1000	(Del Nagro et al., 2014)
Anti-goat	Donkey	Licor IRDye 800CW (925-32214)	1:1000	(Genead et al., 2012)
Secondary antibodies–immunofluorescence				
Anti-rabbit	Donkey	Alexa Fluor 488-conjugated (AbCam Ab150073)	1:500	(Zhang et al., 2014)
Anti-mouse	Chicken	Alexa Fluor 555-conjugated (AbCam150114)	1:500	(Na Pombejra et al., 2017)

Journal of Cell Science • Supplementary information

Figure S1

The antibody used for RAGE immunofluorescent staining of DRG neurons in Figure 2 was verified for specificity in immortalised rat DRG 50B11 neurons. This confirmed that the antibody detected a single band of size ~45kD. B= basal glucose (36mM), H = high glucose (66mM)

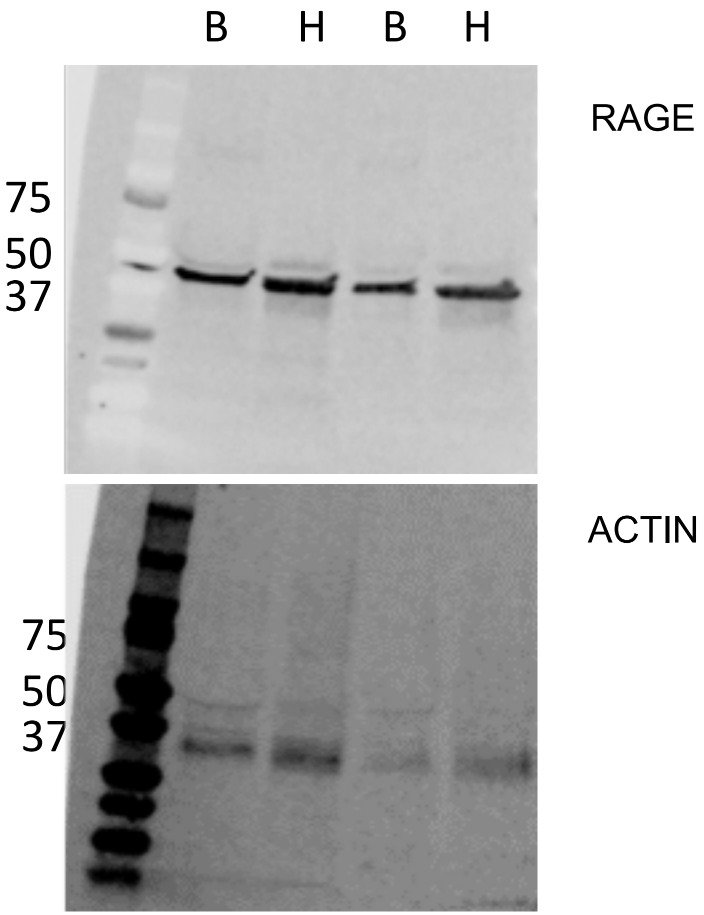

Journal of Cell Science • Supplementary information
